# Profibrinolytic Effect of the Epigenetic Modifier Valproic Acid in Man

**DOI:** 10.1371/journal.pone.0107582

**Published:** 2014-10-08

**Authors:** Ott Saluveer, Pia Larsson, Wilhelm Ridderstråle, Thórdís J. Hrafnkelsdóttir, Sverker Jern, Niklas Bergh

**Affiliations:** 1 The Wallenberg Laboratory for Cardiovascular Research, Institute of Medicine, Sahlgrenska Academy, University of Gothenburg, Gothenburg, Sweden; 2 Department of Cardiology, Landspitali University Hospital and the University of Iceland, Reykjavik, Iceland; Maastricht University Medical Center, Netherlands

## Abstract

**Aims:**

The aim of the study was to test if pharmacological intervention by valproic acid (VPA) treatment can modulate the fibrinolytic system in man, by means of increased acute release capacity of tissue plasminogen activator (t-PA) as well as an altered t-PA/Plasminogen activator inhibitor -1 (PAI-1) balance. Recent data from in vitro research demonstrate that the fibrinolytic system is epigenetically regulated mainly by histone deacetylase (HDAC) inhibitors. HDAC inhibitors, including VPA markedly upregulate t-PA gene expression in vitro.

**Methods and Results:**

The trial had a cross-over design where healthy men (n = 10), were treated with VPA (Ergenyl Retard) 500 mg depot tablets twice daily for 2 weeks. Capacity for stimulated t-PA release was assessed in the perfused-forearm model using intra-brachial Substance P infusion and venous occlusion plethysmography. Each subject was investigated twice, untreated and after VPA treatment, with 5 weeks wash-out in-between. VPA treatment resulted in considerably decreased levels of circulating PAI-1 antigen from 22.2 (4.6) to 10.8 (2.1) ng/ml (p<0.05). It slightly decreased the levels of circulating venous t-PA antigen (p<0.05), and the t-PA:PAI-1 antigen ratio increased (p<0.01). Substance P infusion resulted in an increase in forearm blood flow (FBF) on both occasions (p<0.0001 for both). The acute t-PA release in response to Substance P was not affected by VPA (p = ns).

**Conclusion:**

Valproic acid treatment lowers plasma PAI-1 antigen levels and changes the fibrinolytic balance measured as t-PA/PAI-1 ratio in a profibrinolytic direction. This may in part explain the reduction in incidence of myocardial infarctions by VPA treatment observed in recent pharmacoepidemiological studies.

**Trial Registration:**

The EU Clinical Trials Register 2009-011723-31

## Introduction

Cardiovascular disease is a major cause of death and disability globally. Current preventive antithrombotic treatment options target the coagulation cascade or the platelets. However, the drugs must be dosed at a suboptimal clot-preventing level that leaves the patient with a substantial residual risk, because of the dose-depending risk of severe bleeding complications. In vivo the endogenous fibrinolytic system has an important role in preventing excessive clot formation. The balance between the key activator of fibrinolysis, tissue plasminogen activator (t-PA), and its main inhibitor plasminogen activator inhibitor-1 (PAI-1) is crucial for the potency of the endogenous fibrinolytic system. The vascular endothelium plays a pivotal role in its ability to produce and release t-PA [1], [2], while the main source of plasma PAI-1 is the platelets [3], [4]. The platelets retain high levels of mainly active PAI-1 [5].

Several studies have implied that patients with coronary artery disease (CAD) have impaired fibrinolytic capacity [6], [7]. Impaired acute t-PA release or increased plasma levels of PAI-1 predict future adverse cardiovascular events [8], [9], [10]. Hypertension is also associated with impaired fibrinolytic capacity [11], [12], [13]. This impairment is improved by chronic anti-hypertensive treatment [14], but not by acute blood pressure lowering [15], altogether suggesting a smaller releasable intracellular t-PA pool. Recently, pharmacological inhibition of PAI-1 has shown to protect against hypertension and vascular senescence in mice [16]. However, there is no clinically available PAI-1 antagonist on the market.

Pharmacological tools to directly target the endogenous fibrinolytic system have been lacking. Research by us and others has shown that the t-PA-gene is sensitive to epigenetic control, and several histone deacetylase (HDAC) inhibitors markedly upregulate the t-PA-gene expression in vitro [17], [18], [19]. Amongst all the identified and developed HDAC inhibitors, valproic acid (VPA) is already clinically well-established as one of the most commonly used antiepileptic drugs worldwide [20].

It is of great clinical importance to establish if HDAC inhibitors could be used in man to modulate the endogenous fibrinolytic system. This hypothesis is supported by pharmacoepidemiological studies, where VPA in contrast to other antiepileptic drugs was found to significantly diminish the risk of myocardial infarction in patients with epilepsy compared with controls [21], [22].

This phase IIA study was an open prospective trial with a cross-over design, investigating if treatment with clinically used doses of VPA affects the fibrinolytic system in healthy subjects. The primary endpoint of the study was if VPA induces a profibrinolytic state in man. The fibrinolytic balance was measured as t-PA:PAI-1 antigen ratio as well as the stimulated capacity for acute t-PA antigen release. Secondary effect variables were platelet number and function.

## Methods

### Subjects

Ten healthy, non-smoking white male subjects, aged 50-70 years were recruited by advertisement in a local newspaper. Patients with overt cardiovascular disease, hypertension, blood lipid derangement or diabetes mellitus were not included. The study protocol was approved by the Ethics Committee of the University of Gothenburg (Dnr: 297-09) and by the Medical Products Agency in Sweden (EudraCT Number: 2009-011723-31). The trial was conducted according to the Declaration of Helsinki. The nature, purpose and potential risks of the study were carefully explained to each subject before written informed consent was obtained. The study was performed at The Wallenberg Laboratory for Cardiovascular and Metabolic Research, Sahlgrenska University Hospital, Gothenburg, Sweden. The protocol for this trial and supporting TREND checklist are available as supporting information; see Checklist S1 and Protocol S1.

### Study design

**Figure 1:**
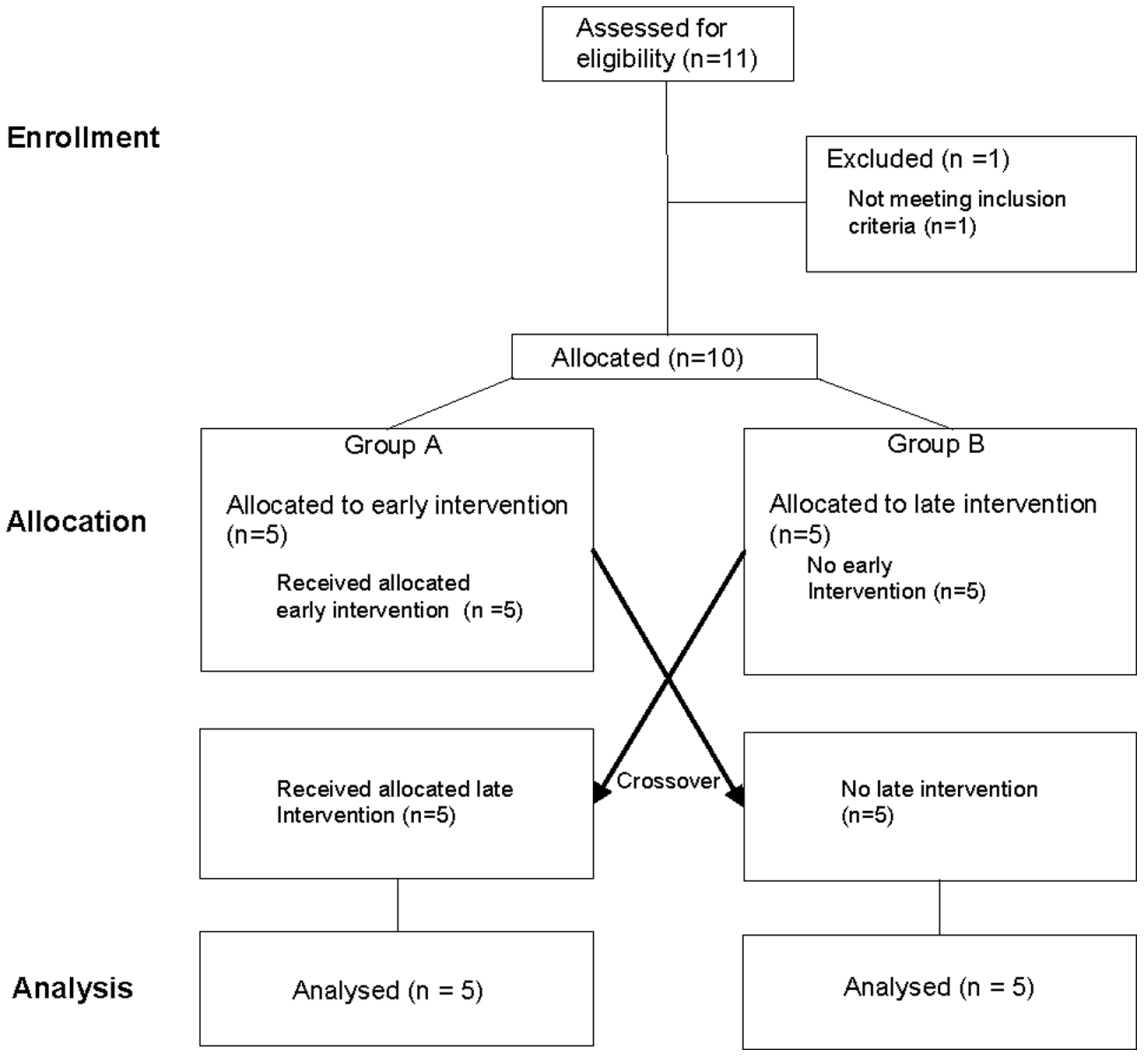
Consort flow diagram.

A baseline physical examination, ECG and analysis of routine blood chemistry were initially performed. After inclusion, the capacity for acute stimulated t-PA release was assessed with the perfused-forearm model [23], [24]. The study had a cross-over design (Figure 1) where the subjects were allocated to group A (n = 5) or B (n = 5). Group A received Ergenyl Retard (Sanofi, 500 mg b.i.d.) for two weeks before the first examination while group B received Ergenyl Retard two weeks before the second examination. There was a wash-out period of 5 weeks between the two perfused forearm experiments. The responsible physician for the study had continuous contact with the study subjects throughout the whole study to minimize the risk for dropouts or reduced compliance.

### The perfused-forearm study

The subjects attended the laboratory after an overnight fast. They had previously been instructed to refrain from coffee for 12 hours and from exhaustive physical exercise and alcohol for 24 hours. A 4 French arterial catheter (Careflow, Becton Dickinson) was introduced percutaneously with modified Seldinger technique into the brachial artery in the non-dominant arm. An indwelling cannula was placed retrogradely into a deep ipsilateral antecubital vein for blood sampling. Intra-arterial blood pressure was recorded by an electrical transducer connected to a SC 9000 monitor (Siemens Medical Systems Inc). After each venous blood sampling, forearm blood flow (FBF) was assessed by venous-occlusion plethysmography with a mercury-in-silastic strain gauge using the MAPPC software (Elektromedicin AB). Means of 3 to 5 recordings were expressed in millilitres per minute and litre of tissue. Intra-observer and inter-observer coefficients of variation in our laboratory have previously been reported to be, on the average, 5.6% and 4.6%, respectively [14]. The laboratory assistant, who analyzed the forearm blood flow curves, was blinded for the treatment status of the subjects.

After cannulation of the artery, 30 minutes were allowed before starting the experiment with a baseline recording for 15 minutes. Thereafter, Substance P (Substance P, Bachem) at the concentration of 8 pmol/mL, was infused for 20 minutes into the brachial artery at a constant rate of 1 mL/min. Post-infusion recordings were performed for 10 minutes. The dose of Substance P was chosen to obtain maximal t-PA release without systemic effects. During pre- and post-infusion baseline periods, blood samples were collected simultaneously from the brachial artery and vein. During Substance P infusion, venous blood samples were obtained at 1.5, 3, 6, 9, 12, 15, and 18 min. To avoid interruption of the infusion, arterial blood was obtained only at baseline and at the end of the infusion and in-between values were interpolated from these values. Blood was collected in chilled, non-vacuated tubes containing 1/10 vol. 0.45 M sodium citrate buffer pH 4.3 (in-house made), for determination of fibrinolytic proteins. Tubes were kept on ice until plasma was isolated by centrifugation at 4°C and 2000 g for 20 min. Plasma aliquots were immediately frozen and stored at −70°C until assay.

### Biochemical analyses

Plasma concentrations of total t-PA antigen and PAI-1 antigen were determined by ELISA (TintElize t-PA, Triolab; Coalize PAI-1, Chromogenix AB) according to manufacturer protocol. Both assays detect free and complexed forms of the respective proteins with equal efficiency, according to the product sheets. The linear correlation coefficient between free and complexed t-PA was 0.99. Samples from one experiment were assayed in duplicate on the same microtest plate. Blood chemistry analyses were performed by standard methods at the Department of Clinical Chemistry at the Sahlgrenska University Hospital. Platelet function was analysed using multiple electrode aggregometry according to manufacturer protocol (Multiplate; Verum Diagnostica GmbH, Munich, Germany). The platelet function was analysed with ADP- (adenosine-diphospate), ASPI- (arachidonic acid), TRAP- (thrombinreceptor peptide) and RISTO-test (ristocetin).

### Calculations

FBF was interconverted to forearm plasma flow (FPF) by hematocrit. Venoarterial concentrations gradients were obtained by subtraction of readings in simultaneously collected venous and arterial samples. Net release or uptake rates for t-PA were calculated as the venoarterial concentration gradient times FPF.

### Statistical analysis

Unless otherwise stated, values are presented as mean and standard error of the mean (SEM). Responses to VPA and Substance P were analysed using paired t-test, 2-way ANOVA (treatment/no treatment and time), one-way ANOVA for repeated measurements as well as the non-parametric Binomial and Related-Samples Wilcoxon Signed Rank Test. Findings were considered significant at p<0.05. All statistical analyses were performed with SPSS (version 18.0, SPSS, Chicago, Illinois).

A pre-study sample size analysis was performed using an estimated effect size of 40% along with a within-group variability of 20% of the total response, power of 0.8 and an α value of 0.05, which provided an estimated sample size of 10.

## Results

11 potential subjects were enrolled in the study after responding to the advertisement in the local newspaper. 10 subjects were included in the trial. The complete data range from participant recruitment to the last visit of the last subject participating in the study was 4^th^ September to 30^th^ November 2011. Baseline characteristics of the study subjects are shown in Table 1. All ten study subjects completed the study. There were no adverse events during the study. The compliance for drug administration was 100%.

**Table 1:** Baseline characteristics of the study subjects (n = 10).

Parameter	Mean (SEM)
Age, years	64.1 (1.4)
Systolic blood pressure, mmHg	137.0 (4.9)
Diastolic blood pressure, mmHg	80.6 (1.8)
Body mass index, kg/m^2^	28.9 (0.9)
Total cholesterol, mmol/L	5.2 (0.3)
LDL cholesterol, mmol/L	3.7 (0.3)
HDL cholesterol, mmol/L	1.1 (0.1)
Triglycerides, mmol/L	1.3 (0.2)
Hemoglobin, g/L	147.4 (3.4)
Creatinine, µmol/L	88.8 (5.0)
Glucose, mmol/L	5.7 (0.2)
High sensitive CRP, mg/L	1.6 (0.3)

Abbreviations: CRP =  C-reactive protein, LDL =  low density lipoprotein, HDL =  high density lipoprotein.

### Hemodynamic responses

**Figure 2:**
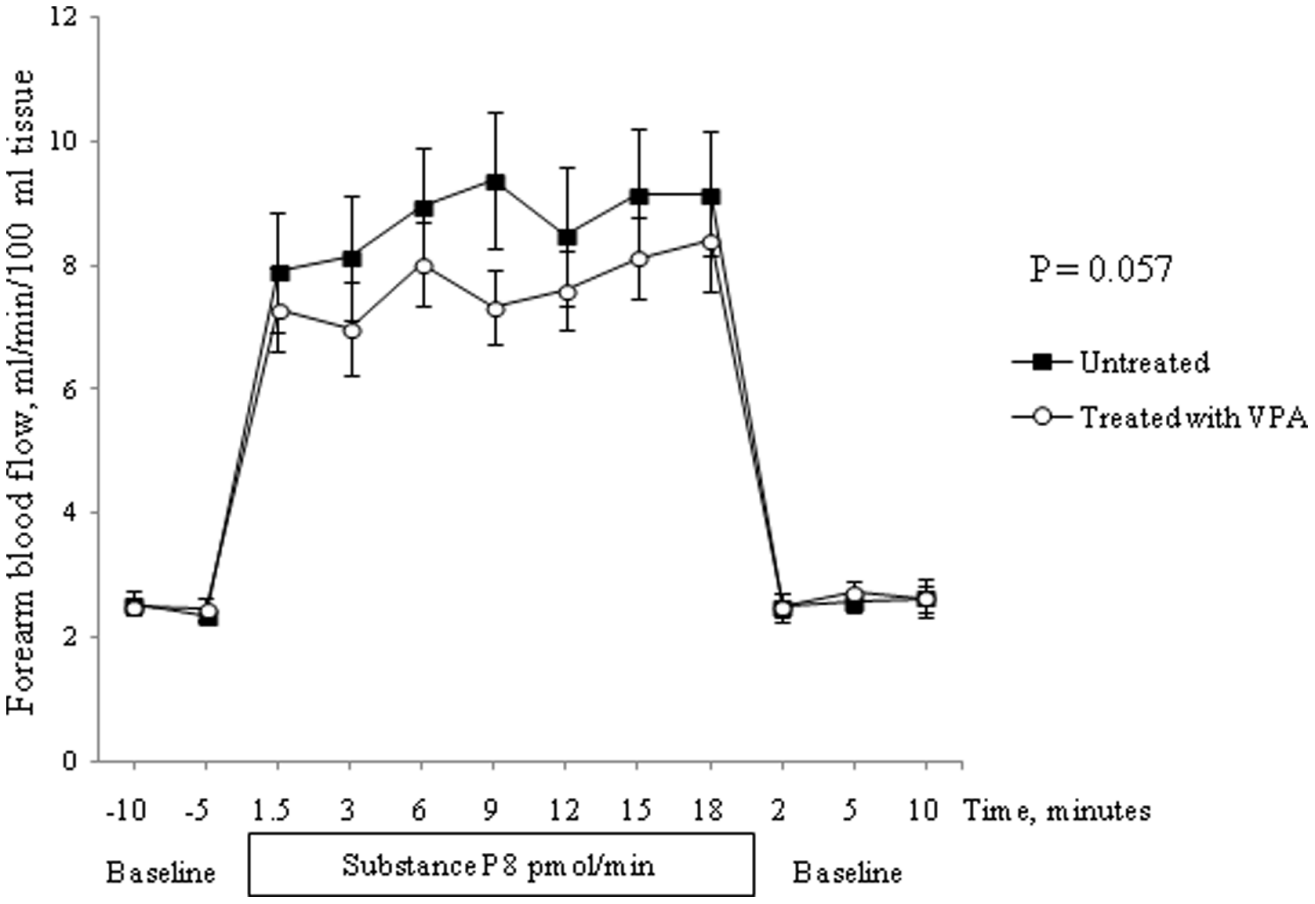
Forearm blood flow during baseline and in response to 20 min infusion of Substance P (8 pmol/min) in untreated (▪) and VPA treated (◯) healthy subjects (n = 10). Baseline measurements 15 min before and 10 min after the infusion. 2-way ANOVA, mean and SEM.

Baseline hemodynamic and fibrinolytic variables before the two infusion studies are shown in Table 2. Baseline FBF was similar in VPA treated and untreated patients (p = ns, t-test, Table 2). Systolic and diastolic blood pressure levels were unaffected by VPA (p = ns, t-test). Intra-brachial Substance P infusion resulted in a significant increase in FBF on both occasions (p<0.0001 for both, ANOVA). FBF responses to Substance P infusion showed a tendency to suppression after VPA treatment, compared to untreated subjects (p = 0.057, 2-way ANOVA, Figure 2).

**Table 2:** Study parameters.

Parameter	Before VPA	After VPA	P-value
Serum valproate, µmol/L	0	426 (25)	<0.0001
Baseline FBF, ml/min/100 ml tissue	2.84 (0.19)	2.73 (0.18)	ns
Baseline venous t-PA antigen, ng/ml	10.54 (0.65)	8.57 (0.41)	<0.05
Baseline t-PA release, ng/min/L tissue	2.16 (3.3)	3.27 (1.4)	ns
Baseline venous PAI-I antigen, ng/ml	22.2 (4.6)	10.8 (2.1)	<0.05
Baseline venous t-PA:PAI-1 ratio	0.74 (0.17)	1.03 (0.17)	<0.01
Baseline arterial t-PA:PAI-1 ratio	0.85 (0.17)	1.19 (0.20)	<0.01

### t-PA baseline levels and responses

VPA decreased baseline venous t-PA antigen levels by approximately 20% (p<0.05, t-test, Table 2). In 9 of 10 patients baseline t-PA antigen decreased after VPA treatment (p = 0.02, and p = 0.01, Binomial Test and Related-Samples Wilcoxon Signed Rank Test, respectively). Baseline t-PA release was 2.16 (3.3) and 3.27 (1.4) ng/min/L tissue during untreated and VPA treated conditions, respectively (p = ns, t-test).

**Figure 3:**
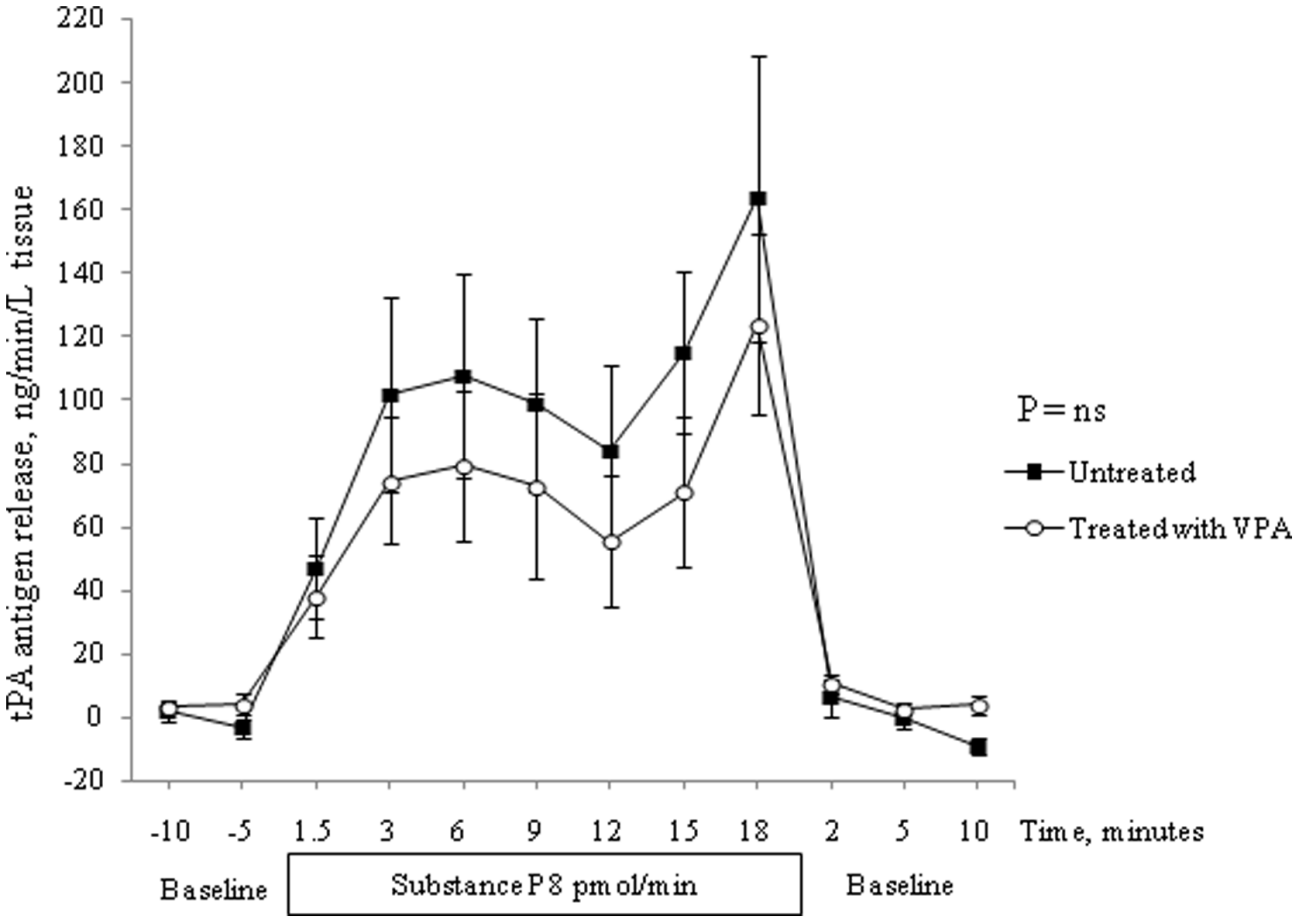
Net forearm release rates of t-PA antigen during baseline and in response to 20 min infusion of Substance P (8 pmol/min) in untreated (▪) and VPA treated (◯) healthy subjects (n = 10). Baseline measurements 15 min before and 10 min after the infusion. 2-way ANOVA, mean and SEM.

The t-PA antigen release in response to intra-brachial Substance P infusion was not affected by VPA treatment (p = ns, 2-way ANOVA, Figure 3). The average time to peak t-PA secretion in untreated and treated subjects was 13.2 (1.7) and 11.0 (2.1) minutes, respectively (p = ns, t-test).

### Plasminogen activator inhibitor-1 levels

**Figure 4:**
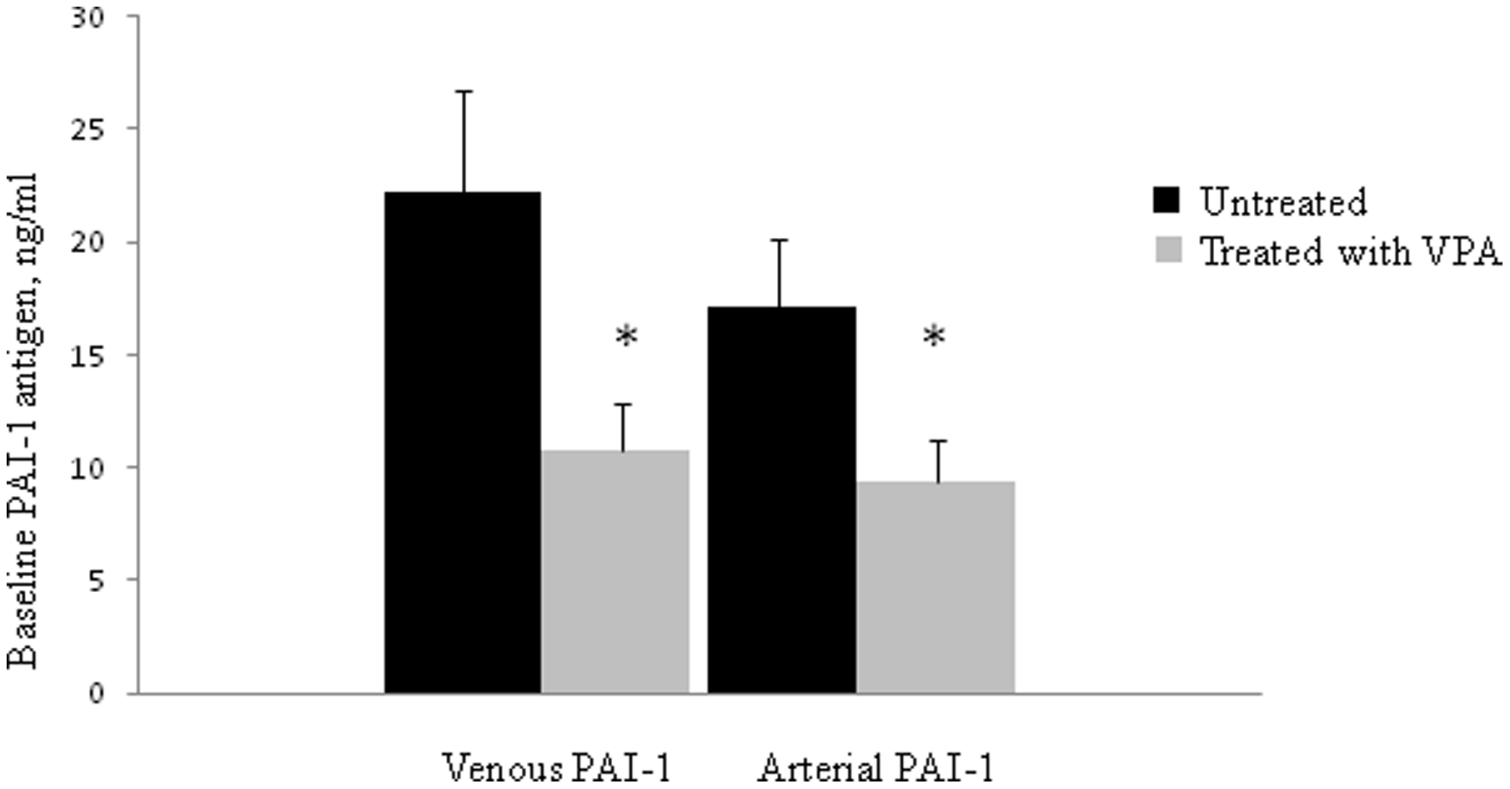
Baseline venous and arterial plasminogen activator inhibitor-1 (PAI-1) antigen levels in untreated (▪) and VPA treated (grey square) subjects. *p<0.05, paired t-test, untreated versus treated.

Circulating plasma levels of PAI-1 were significantly reduced from 22.2 (4.6) to 10.8 (2.1) ng/ml after VPA treatment (p<0.05, t-test, Figure 4). In all 10 patients baseline venous PAI-1 antigen decreased after VPA treatment (p = 0.002, and p = 0.005 for Binomial Test and Related-Samples Wilcoxon Signed Rank Test, respectively). During substance P infusion, there was no detectable release of PAI-1 from the forearm, either in the treated or untreated condition (data not shown, p = ns).

### Fibrinolytic balance

The ratio of baseline venous t-PA:PAI-1, and baseline arterial t-PA:PAI-1 antigens were both significantly increased by VPA treatment (p<0.01 for both, t-test, Table 2).

### Platelet count and aggregation tests

VPA treatment did not alter the platelet counts (p = ns). Platelet aggregation responses were evaluated in whole blood samples using Multiplate analysis induced by ASPI, ADP, TRAP, and RISTO-low and -high, respectively. Platelet aggregation was not affected by VPA-treatment (data not shown, p = ns).

## Discussion

In the present study we examined if treatment with valproic acid affects the regulation of the main enzymes of the fibrinolytic system in healthy man. Our main finding was markedly reduced circulating plasma PAI-1 antigen levels after VPA treatment. The capacity for acute stimulated t-PA antigen release was not affected. By contrast, basal levels of t-PA and PAI-1 antigen were reduced by 19% and 51%, respectively. This may indicate that the fibrinolytic balance between t-PA and PAI-1 antigen levels was altered, resulting in a profibrinolytic state in man.

Our finding of diminished circulating levels of PAI-1 antigen after VPA treatment is very interesting and might translate into decreased cardiovascular risk, as PAI-1 is the main inhibitor of active t-PA, and thereby of vascular fibrinolysis [25]. Angiotensin-converting enzyme inhibitors (ACE-I) have also been shown to have a suppressive effect on PAI-1 levels, altering the fibrinolytic balance in favor of fibrinolysis [13]. Results from large placebo-controlled trials regarding patients with ventricular dysfunction after myocardial infarction have shown a significant decrease in the incidence of coronary events in patients treated with ACE-I, irrespectively of its effect on the blood pressure [13]. The favorable impact of ACE-I on the fibrinolytic balance might contribute to the observed cardioprotective effect. However, no specific study exists indicating that drugs that improve the fibrinolytic balance are superior in terms of reduction in stroke or coronary events, although indirect evidences support this possibility. Further studies are necessary to elucidate the clinical impact of an improved fibrinolytic balance as well as the mechanism behind the down-regulatory effect of VPA on PAI-1 levels. Epigenetic regulatory mechanisms on PAI-1 expression have not been studied so far in humans. In vitro clinical doses of VPA did not have any effect on PAI-1 gene transcription in human umbilical vein endothelial cells [17]. However, in an in vivo model in mice we have observed a suppression of PAI-1 mRNA in aortic endothelial cells after one week pretreatment with VPA (unpublished data).

A well known side effect of VPA is thrombocytopenia but we did not observe any suppression of platelet count or function after 2 weeks of VPA treatment (data not shown). Previous studies have shown small effects on platelet count only in a minority of patients after long-term treatment [26]. Thus, the diminished PAI-1 after VPA cannot be explained by reduced platelet number.

We have previously demonstrated a marked upregulation of the t-PA-gene expression by VPA in vitro mainly through its HDAC inhibitory effect [17], [18]. The upregulation in cultured human endothelial cells was reflected by both increased mRNA and protein levels. This effect was established within 24 hours and was observed in human endothelial cells from coronary arteries, aorta and the umbilical vein. The upregulation of t-PA was 10–15 fold compared to basal levels for both mRNA and protein at a concentration of 3 mM of VPA in the cell culture medium. However, the effect on t-PA-gene expression was highly concentration-dependent, and within the therapeutic range used in the clinical practice for prevention of epilepsy seizures we observed a 2–4 fold induction of t-PA in vitro [17]. However, to extrapolate the optimal concentration from in vitro to in vivo is complex and requires further studies. In this study the mean serum valproate concentration in the treated subjects was 426 µmol/L, which is in the lower part of the therapeutic concentration range (300–700 µmol/L). Further studies will have to elucidate the optimal dose as well as treatment duration effects on the fibrinolytic balance.

Most of the t-PA in plasma is complex bound to PAI-1 during baseline conditions [27]. It is only the free fraction of t-PA that is enzymatically active. There is a strong association between PAI-1 and t-PA antigen levels [28]. A possible explanation for this is that the hepatic clearance of t-PA/PAI-1 complex is slower than for free t-PA. Thus, the hepatic clearance of total t-PA is inversely related to PAI-1 antigen levels, and basal t-PA antigen level is in part a surrogate measure of PAI-1 level [28]. This could explain our finding of decreased t-PA antigen at baseline, as a result from diminished PAI-1 antigen resulting in a reduced fraction of t-PA/PAI-1 complexes.

Both t-PA and PAI-1 levels are risk factors for a first myocardial infarction [9], [29], [30], and they are also significant risk markers for recurrent myocardial infarction [31], [32]. It is not obvious, however, why elevated t-PA antigen levels are associated with coronary events, since t-PA is the key profibrinolytic enzyme. We have previously demonstrated that baseline venous plasma t-PA (or PAI-1) does not predict local fibrinolytic capacity [33], and it is likely that it is the local t-PA release capacity that is essential for the protection against occlusive thrombus formation. There are alternative explanations why systemic t-PA is a cardiovascular risk factor. First, baseline t-PA antigen might be reflective of the amount of PAI-1 [30]. The t-PA levels may also reflect the acute phase response in CAD or may indicate endothelial dysfunction or net activation of the fibrinolytic system due to underlying atherosclerosis [9].

Results from two recent pharmacoepidemiological studies suggest that VPA may have beneficial cardiovascular effects, as it was found to diminish the risk of myocardial infarction and stroke in large cohorts [21], [22]. A possible explanation to the observed cardiovascular protection by VPA might be related to the diminished levels of PAI-1 and the beneficial fibrinolytic balance, measured as t-PA:PAI-1 ratio in the present study.

There are several limitations of the study. First, we have only stimulated the endothelium with Substance P, and it still remains unknown if another physiological endothelium agonist could have led to increased stimulated t-PA release capacity. Second, the healthy subjects in this study might have a normal t-PA production and release capacity from the beginning, and VPA cannot be expected to increase the intracellular t-PA pool to supra-physiological levels. Third, as VPA may decrease the expression of endothelial nitric oxide synthase [34], we observed a tendency to impaired endothelium-dependent vasodilation after VPA. This could have influenced the measurements of stimulated t-PA release capacity. Fourth, fibrinolytic function was measured in this study as dependent on t-PA and PAI-1 antigen levels in plasma. It would be of value to have a more functional assay. However, since the endogenous fibrinolytic system is dependent on the acute release of t-PA upon stimulation from the local endothelial cell layer, functional assays such as clot lysis, thrombo-elastogram are probably not relevant for testing the hypothesis. Finally, the perfused forearm model might not be the optimal means to assess the local fibrinolytic capacity in healthy subjects, where effects on the fibrinolytic system could be more difficult to prove locally in the forearm model compared to the systemic level. Further studies are needed to elucidate the mechanism behind the improved fibrinolytic balance as well as studies in patients with defect endogenous fibrinolytic system. There is a possibility that patients with a defect fibrinolytic system may respond differently to VPA treatment than healthy controls. It may be easier to normalize an impaired t-PA production compared with inducing supra-physiological levels of intracellular t-PA.

## Conclusion

Treatment with the epigenetic modifier valproic acid did not affect the local stimulated t-PA release capacity from forearm in healthy men. On the systemic level, however, valproic acid significantly decreased plasma levels of PAI-1 antigen, and altered the balance between t-PA and PAI-1 antigen. This may cause a profibrinolytic state in man. The divergent results might depend on the cohort of apparently healthy men, where effects might be more difficult to show locally than systemically. Further studies are motivated to evaluate the effects of valproic acid or other histone deacetylase inhibitors on the fibrinolytic system in patients with impaired capacity for endogenous fibrinolysis, e.g. in patients with manifest atherosclerosis.

## Supporting Information

Checklist S1 **TREND Checklist.**
(PDF)

Protocol S1 **The study protocol.**
(DOCX)
