# Supplementary material for: Profibrinolytic Effect of the Epigenetic Modifier Valproic Acid in Man
Source: PLoS One. 2014 Oct 8;9(10):e107582. doi: 10.1371/journal.pone.0107582 (PMC4189785; doi:10.1371/journal.pone.0107582)
Supplement: Protocol S1 — The study protocol. (DOCX) [file pone.0107582.s002.docx]

# Effect of valproic acid on fibrinolysis in man

### Study protocol

**Aim:**

To investigate if treatment with valproic acid results in increased t-PA release from the endothelium and improved endogenous fibrinolytic balance in man.

**Background:**

Tissue-type plasminogen activator (t-PA) is a protein that starts thrombolytic mechanisms and is thereby involved in processes which prevents the formation and dissolves thrombosis in blood vessels. t-PA-activity is regulated by inhibitors, whereby the most important is PAI-1. The cells which form the innermost layer in the wall of blood vessels (endothelial cells), produce t-PA and continuously release a smaller amount of the protein to blood circulation. Because t-PA is also stored in the endothelial cells, stimulation of the endothelium can result in more that 20-fold increase of the t-PA release with a powerful increase of the t-PA concentration near the thrombus.

Our research group has since 1994 used an experimental set-up, ”the perfused forearm model”, where the capacity of t-PA release can be studied in humans. Among other results, we have found that patients with hypertension, and patients with renal dysfunction (these patients have increased risk of cardiovascular disease), have markedly decreased capacity of stimulated t-PA release.

Furthermore, our studies have demonstrated that treatment of hypertension improves the fibrinolytic capacity but the mechanisms involved are uncompletely mapped. The problem has been that there has not been any tool to stimulate the t-PA synthesis.

Our research group has recently found that valproic acid (VPA), which is a well-established drug for treatment of patients with epilepsia, dramatically upregulates the tPA-gene expression in endothelial cell culture and results in both increased mRNA and protein level. This upregulation occurs within 24 hours and is seen in human endothelial cells from coronary arteries, umbilical vein and aorta. Upregulation of t-PA is in size of 10-15 fold increase compared with basal levels for both mRNA and protein. The concentration of VPA used in the cell culture medium, is on the same level as can be measured in the blood of patients with treated epilepsia, i.e. therapeutic levels. It is very important to establish if upregulation of t-PA also occurs in man; in that case we could possibly have a potent drug for stimulation of the endogenous fibrinolysis.

The most important effect variables in the study is t-PA release, PAI-1 levels, changes in fibrinolytic balance and other measures of blood vessel function in terms of blood flow changes during stimulation. The study is expected to increase the knowledge about regulation of human defense mechanisms against thrombosis formation via newly discovered regulatory mechanisms.

**Study design:**

Prospective hypothesis testing trial investigating effects on the endogenous fibrinolytic system before and after VPA treatment (effects after initiation and after drug withdrawal).

Sixteen healthy volontary male persons, aged 50-70 years, are included after advertisements.
Exclusion criteria: smoking, female sex, hypertension, epilepsia, BMI> 35 kg/m², diabetes, established cardiovascular disease, malignancy, psychiatric disorder, alcoholism, all forms of chronic disease, acute infection and inflammation.

After inclusion, the subjects are investigated with an invasive test at 2 occasions (Study day I and II) regarding the ability to release t-PA, before and after VPA treatment.

After the initial visit, the subjects are randomized to one of two treatment groups. One group receives 2 weeks of VPA treatment prior to Study day I, and the other group receives 2 weeks of VPA treatment prior to Study day II.

The subjects are investigated regarding the capacity to release t-PA with an invasive test, using the perfused forearm model in the non-dominant arm. The release is stimulated by intra-arterial infusion of Substance P. For them who received treatment first, the drug is withdrawn. After 3 weeks of wash-out without medication, the subject switches treatment group (VPA alternatively no treatment for the following 2 weeks). Thereafter, a new perfused forearm study is performed and any treatment is withdrawn. The time between Study day I and II is 5 weeks.

**Accomplishment:**

The study is expected to start under the autumn of 2011 and will continue during 1/2 year.

**Asking for participation:**

Healthy volontary men are recruited via local advertisements. Subjects who have declared their interest, will be contacted and an individual preparing visit will be scheduled.

**Preparing visit:**

The subject gets oral and written information about the study. A clinical examination is performed (see protocol), and basal blood samples are taken according to protocol. After written informed consent is obtained, the Study day I is booked. The following blood samples are taken:

- Hb, Lpk, tpk
- Na, K, Krea, hsCRP, glukos
- ASAT, ALAT, ALP, Bilirubin, PK, APTT
- Cholesterol, LDL, HDL, Triglycerids (fasting on Study day I)

Thereafter, the subject is randomized to either early VPA treatment (before Study day I) or late treatment (before Study day II). VPA-treatment: Depot tablet Ergenyl Retard 500 mg, 1 tablet twice daily for 2 weeks. The subject starts medication in the evening, 2 weeks prior to Study day I or II. After 2 weeks of treatment, the last tablet is taken in the morning, in relation to Study day I or II.

**Generally before Study day I-II:**

No intake of tea or coffee in the morning. For 7 days before the experiments,the subject is not allowed to use vitamins or aspirin. No NSAID’s the day before the experiment or during the study day. If required, analgetics in form of paracetamol. Food/drink that contains coffeine or theophylline (bananas, coffee, tea, chocolate) is not allowed for 12 hours before the experiment. No alcohol for the last 24 hours. The subject arrives at the laboratory in the morning at 08:00 A.M., fasting since midnight. Exposition of checklist before each experiment.

Blood concentration of valproate is controlled in all subjects during Study day I and II.

The rest of blood samples controlled in the beginning of Study day I: fB-glucose, cholesterol, LDL, HDL, triglycerider.

**Study day I:**

**The perfused forearm experiment**

See schedule for invasive experiment. The subject is expected to be lying down and relax and rest during the experiment, but is allowed to listen to music or radio if wished. Venous cannulas are inserted into deep antecubital veins in both arms. Arterial catheter is inserted into the a. brachialis in the non-dominant arm. Equipment for plethysmography is connected to both arms for measurements of local blood flow. Blood concentration of valproate is tested in all subjects.

When all the equipment is connected, the subject is going to rest for 30 minutes before the experiment starts. The arrangement is as follows: blood samples are taken simultaneously from arterial and venous catheters and basal blood flow is registered to evaluate the basal t-PA release and blood flow. Thereafter starts an intra-arterial infusion of Substance P, 8 pmol/ml, 1 ml/min. Blood flow measurements and blood samples are gathered repetitively during the 20 minute long infusion to evaluate the t-PA-release, PAI-1 levels and the blood flow increase in the forearm. During the following 10 minutes after stopped infusion, basal blood samples are measured again to follow the tPA-release after the course. After finished examination all the equipment is removed. The venous catheters are removed. At last the arterial catheter is removed and the puncture site is compressed for 20 minutes by the physician in charge.

Total time for the experiment is approximately 3 hours including preparations. See the experiment schedule and the blood sample schedule for the invasive forearm experiments.

**Study day II:**

5 weeks after Study day I, the patient will return for study day II. This is accomplished in the same way as study day I. Blood concentration of valproate is controlled in all subjects. The study drug is discontinued in subjects who received the treatment.

**Effect of valproic acid on fibrinolysis in man**

**Schedule for invasive experiment**

**Before the experiment:**

1 Measurement of the forearm volume

2 Checklist

3 Venous cannula in the dominant arm

4 Blood samples: fB-glukos, lipids (study day I), S-valproate (study day I and II)

5 Catheterisation of the non-dominant arteria brachialis

6 Venous cannula in the non-dominant arm

7 ECG and ia pressure is connected

8 Plethysmography is connected according to SOP

9 Rest, approximately 30 min after catheterization

10 Experiment is executed, according to the protocol

**Schedule for blood samples during the invasive test:**

**Baseline 1: 15 min:** Blood samples at 5 och 10 min, **2** **ml art and ven**

**Provocation with Substance P, 8 pmol/ml/min during 20 min:**

Blood samples 2 ml **ven** vid 1.5, 3, 6, 9, 12, 15, and 18 minutes and **2 ml art** at 20 minutes.

**Baseline 2: 10 min:** Blood samples **2 ml** at 2, 5 and 10 min, both **art** and **ven**

During all occasions, 2 ml blood is discarded before blood sample. 2 ml with Stabilyte® 1/10 in the tubes. Flush 2 ml NaCl 0,9%. **Plethysmography, MAP and HR** is registered directly after the blood sample is taken

**After performed forearm experiment**

1 ECG/plethysmography is disconnected. Venous cannula is removed. Arterial catheter is removed, ≥20 min compression of the puncture site, according to SOP.

2 The subject gets information about the experiment and possible actions in case of any complication.

3 The catheterisation protocol is filled in.

**In case of complications**

1 Complications during catheterization are reported in the protocol. In case of more serious complications the physician responsible for the method will be informed and will take any required action.

2 Adverse events related to the drug will be reported to the Swedish Medicinal Agency in relevant cases.

**Effect of valproic acid on fibrinolysis in man**

#### Schedule for dilution

**Substance P, 50µg/ml = 37 µmol/ml**

**Stem solution:**

**1,0** **ml** of 37 µmol/ml Substance P + 99,0 ml NaCl 🡪 370 pmol/ml

2 ml **stem solution** + 90 ml NaCl -------------> 8 pmol/ml

**Effect of valproic acid on fibrinolysis in man**

Personal data to be filed:

Allocation number:_______________________

Name:_________________________________________________

Personal nr:__________________________

**Address:______________________________________________________

Phone number:_____________________________________________

Profession:________________________________________________________


Date for experiment:_____________________


Responsible for investigation:________________________**
**Effect of valproic acid on fibrinolysis in man**

**CRF**

Allocation number:________________ Age:______________

Medicinal history:

Heredity (hypertension, cardiovascular disease, diabetes, venous thromboembolism)? No Yes,__________________

Other diseases? No

Yes,___________________

________________________________________________________________

Drug allergies? No

Yes,___________________________

Previous smoker? Yes No

___________________________________________________________________

Status:

Length:_____m Weight:______kg BMI: ______kg/m2

General state of health:___________________________________________________________________

Heart:_______________________________ Heart frequency:______

Blood pressure:____/____

Lung auscultation:___________________________________________________________

Stomach:________________________________________________________________

Examiner:________________________________Date:___________________

**Effect of valproic acid on fibrinolysis in man**

Checklist before the experiments:

Study day nr: ___________

Date:_________________

Allocation number:_______

Written informed consent? No [ ] Yes [ ]

(Must be „Yes“ before continuing the participation)

Fulfilled inclusion criteria? No [ ] Yes [ ]

Forearm volume:_____________ml

Time for start according to protocol:______________

Blood pressure:___________

Yes No

Medicines? [ ] [ ]

Aspirin/NSAID since 10 days? [ ] [ ]

Fasting after midnight? [ ] [ ]

Followed food recommendation? [ ] [ ]

Coffee/tea last12 h? [ ] [ ]

Vitamins last 10 days? [ ] [ ]

Alcohol last 24 h? [ ] [ ]

Exhaustive fysical activity last 24 h? [ ] [ ]

Recent infection? [ ] [ ]

Do you feel healthy? [ ] [ ]

Catheterization OK? [ ] [ ]

Did you forget any tablets? [ ] [ ]

Have you experimented any side effect? [ ] [ ]

How do you feel? _______________________

Commentary:_______________________

____________________________________

____________________________________

Responsible physician for the experiment:
